# A Rare Incidence of Necrotizing Lesion of the Upper Arm Due to Pentazocine Injection Abuse: A Case Report

**DOI:** 10.7759/cureus.27046

**Published:** 2022-07-20

**Authors:** Seema Yelne, Mayur B Wanjari, Pratiksha K Munjewar, Krutika Malu, Roshan Umate

**Affiliations:** 1 Critical Care, Shalinitai Meghe College of Nursing, Datta Meghe Institute of Medical Sciences (Deemed to be University), Wardha, IND; 2 Epidemiology and Public Health, Jawaharlal Nehru Medical College, Datta Meghe Institute of Medical Sciences (Deemed to be University), Wardha, IND; 3 Medical Surgical Nursing, Smt. Radhikabai Meghe Memorial College of Nursing, Datta Meghe Institute of Medical Sciences (Deemed to be University), Wardha, IND; 4 Dental Surgery, Sharad Pawar Dental College, Datta Meghe Institute of Medical Sciences (Deemed to be University), Wardha, IND; 5 Research and Development, Datta Meghe Institute of Medical Sciences (Deemed to be University), Wardha, IND

**Keywords:** dependence., opiate agonist, paramedics, pentazocine, drug abuse

## Abstract

A patient with a history of treated pain with intravenous drugs comes at high risk for drug abuse. Administration of intravenous pentazocine continuously for a long time in the same location affected the skin tissue. Pentazocine misuse is frequent among patients with chronic disease conditions and the easy availability of pentazocine injection can easily lead to significant consequences. We present a unique case of pentazocine misuse resulting in significant skin necrosis. We present a case of a 48-year-old male with a complaint of pain and wound with lesions over the arm due to the regular self-administered pentazocine in the same location. The therapeutic intervention given in this case is an opiate agonist to control the dependence on pentazocine.

## Introduction

Pentazocine is a morphine synthetic benzomorphan derivative used to treat moderate to severe pain. It is a low-economical mixed agonist-antagonist drug. Its agonist activity mediates pentazocine's analgesic effects at the k-opioid receptor [1]. As per the current literature, pentazocine consumption ranges from 0.1% to 21.8% in various districts of India [2].

Pentazocine produces a high psychological and physical reliance, culminating in a painful withdrawal symptom when the drug is discontinued Abusers first prefer the intravenous route because its substance immediately reaches the brain [3]. Repetitive injections, non-adherence to aseptic techniques, or made by anyone who is not a health professional could lead to vascular and soft tissue damage [4].

Despite the multiple challenges connected with drug injection, most self-injectors do not look for medical attention because of a lack of interest and apathy, embarrassment, drug preoccupation, or severe withdrawal that is alleviated by injecting the substance itself, particularly in the opioid group [5].

## Case presentation

A 48-year-old male came to the casualty department complaining of pain and wound with infection lesions on the left and right brachium regions. The patient reported a previous episode of general body myalgia, for which he reported to a general practitioner around where he lives. At that time, he was prescribed an injection of pentazocine by the local practitioners. The patient got relief after the injection. After that the patient continued injecting pentazocine in both arms frequently on his own as he felt good after each injection. The doctor administered pentazocine to the patient for the pain but the patient was taken regularly and that lead to the abuse stage.

**Figure 1:**
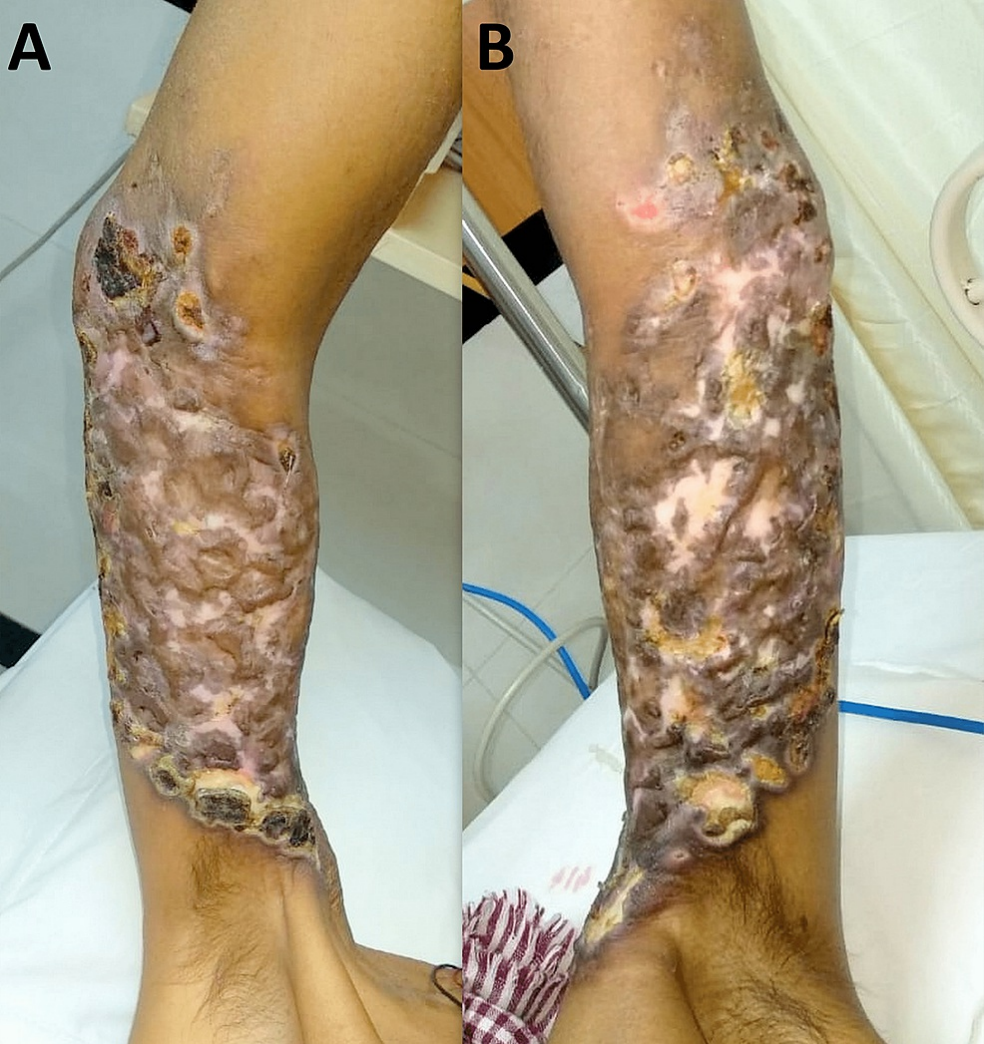
(A, B) Right and left brachium region necrotizing and infection with oozing of the fluid from the lesion.

Physical examination revealed a lesion with necrotizing and infection with oozing of the fluid from the lesion. The erythematous papulonodular lesion with hyperpigmentation around the ulcer and skin contracture on right and left hands saw (Figures 1A, 1B).

Laboratory investigation of the hemoglobin, blood sugar level, kidney and liver function test, and urine investigation were in the normal range. The patient's mental status examination found that he has no psychological or behavioral condition; he is currently in the motivational cycle's preparation stage. The opiate agonist therapeutic intervention was given to the patient to control the dependence on pentazocine and reduce complications. He received motivational treatment and was advised to use a rehabilitation strategy. On regular follow-up, the patient remained abstinent for six weeks but was then lost to follow-up.

## Discussion

Being a synthetic opioid, pentazocine is a narcotic by definition and is rising in India and other developing countries. Pentazocine is commonly abused intravenously, subcutaneously, and intramuscularly [6]. The current case presentation has had several features and noted the abuse of pentazocine dependence in common people with no medical background. The lesions on the patient's upper arms show blind dating, a desperate attempt by the addict to self-administer in the same area on both upper arms. This develops following the onset of cutaneous complications. The necrotizing lesion on the upper arms shows the erythematous papulonodular lesion and hyperpigmentation around the ulcer and skin contracture. The treatment modality used in this case is an opiate agonist to control the dependence on pentazocine.

Worldwide prescription drug abuse is a major health complication that includes various drug-like analgesics and cough syrup being used by the individual for a reason other than the medical indication. The use of opioids such as pentazocine is abused increasingly reported worldwide including in India [7].

Since there have been only a few studies, on pentazocine abuse due to the decrease in its use in developed countries. But it remains a significant problem for an underdeveloped country like Nigeria; physicians continue to prescribe pentazocine to manage acute and chronic pain. A recently published case report addressed a similar case in which sickle cell in young adults developed a dependence on the pentazocine prescribed by the doctors for pain management in hospital settings. Skin ulceration from the combined effects of cellular cytotoxicity and vascular thrombosis may occur when applied via the typical technique known as “skin popping.” Additionally, cocaine's vasoconstrictive impact generates a milieu that is vulnerable to infection and may even work in concert with bacterial proteases to cause necrosis [8].

## Conclusions

Pentazocine abuse is becoming more common worldwide, particularly in developing countries. At various times the doctors prescribe medicine for medical purposes, but that drug patient takes it continuously without permission from the doctors. The clinician should take caution when prescribing pentazocine to the patient. The use of pentazocine is growing with the cause of cutaneous complications. Even if the patient does not provide a history of injection drug use, a localized mark on the skin can give vital hints and alert the doctor to the probability of injectable drug usage, allowing the physician to make early efforts to provide corrective measures. Many impoverished countries have unrestricted over-the-counter access to these drugs; much work is required to regulate their widespread availability. A medical professional plays a critical role in conveying information concerning its addictive properties, intravenous difficulties, and severe consequences.
